# Increased maternal and neonatal morbidity and mortality due to severe acute respiratory syndrome in the early years of COVID-19: a descriptive-analytical study, Rio de Janeiro state, Brazil, 2018-2021

**DOI:** 10.1590/S2237-96222025v34e20240665.en

**Published:** 2025-08-11

**Authors:** Allan Scharf, Danielle Pereira Paulo, Giselle Ferreira de Paes, Welington Lugão, Danielle Coelho de Azevedo, Beatriz Barde, Nádia Rodrigues

**Affiliations:** 1 Universidade Federal de Alagoas, Instituto de Ciências Biológicas e da Saúde, Maceió, AL, Brazil; 2 Universidade do Estado do Rio de Janeiro, Instituto de Medicina Social, Rio de Janeiro, RJ, Brazil

**Keywords:** Pandemics, COVID-19, Pregnant People, Infant, Newborn, Epidemiology, Descriptive, Pandemias, COVID-19, Personas Embarazadas, Recién Nacido, Epidemiología Descriptiva

## Abstract

**Objective:**

To investigate morbidity and mortality rates from severe acute respiratory syndrome in pregnant women and newborns in the first two years of the COVID-19 pandemic.

**Methods:**

This was a descriptive-analytical study in which data on cases and deaths in pregnant women and newborns with a positive diagnosis of severe acute respiratory syndrome, regardless of the etiological agent, were collected from the Influenza Epidemiological Surveillance Information System and data on the number of live births from the Live Birth Information System. The frequencies of cases and deaths and the morbidity and mortality rates among pregnant women and newborns in the state of Rio de Janeiro were calculated using the Kruskal-Wallis test to compare the periods 2018-2019 and 2020-2021.

**Results:**

During the pandemic, there was a statistically significant increase in morbidity rates in pregnant women (24.49/100,000 to 537.39/100,000; p-value<0.001) and newborns (69.97/100,000 to 200.82/100,000; p-value 0.022) and mortality rates in pregnant women (1.87/100,000 to 48.08/100,000; p-value<0.001) and newborns (1.87/100,000 to 9.26/100,000; p-value 0.008).

**Conclusion:**

This study identified a statistically significant increase in morbidity and mortality rates due to severe acute respiratory syndrome in pregnant women and newborns in the state of Rio de Janeiro during the pandemic. There is a clear need for greater investment to reduce harm to these groups, such as vaccination and access to the healthcare system, as well as highly complex infrastructure throughout the state.

## Resumen

**Objetivo:**

Investigar las tasas de morbilidad y mortalidad por síndrome respiratorio agudo grave en mujeres embarazadas y recién nacidos durante los dos primeros años de la pandemia de COVID-19.

**Métodos:**

Se trata de un estudio descriptivo-analítico en el que se recopilaron datos de casos y muertes en mujeres embarazadas y recién nacidos con diagnóstico positivo de síndrome respiratorio agudo grave, independientemente del agente etiológico, en el Sistema de Información de Vigilancia Epidemiológica de la Gripe, y datos del número de nacidos vivos en el Sistema de Información sobre Nacidos Vivos. En las gestantes y los recién nacidos, se calcularon las frecuencias de casos y muertes y las tasas de morbilidad y mortalidad mediante la prueba de Kruskal-Wallis en el estado de Río de Janeiro para comparar los periodos 2018-2019 y 2020-2021.

**Resultados:**

Durante la pandemia, se observó un aumento estadísticamente significativo de las tasas de morbilidad en embarazadas (24,49/100.000 a 537,39/100.000; p-valor<0,001) y neonatos (69,97/100.000 a 200,82/100.000; p-valor 0,022) y de las tasas de mortalidad en gestantes (1,87/100.000 a 48,08/100.000; p-valor<0,001) y neonatos (1,87/100.000 a 9,26/100.000; p-valor 0,008).

**Conclusión:**

Este estudio identificó un aumento estadísticamente significativo en la pandemia de las tasas de morbilidad y mortalidad por síndrome respiratorio agudo grave en gestantes y neonatos en el estado de Río de Janeiro. Se destaca la necesidad de una mayor inversión para reducir los daños en estos grupos, como la vacunación y el acceso al sistema de salud, y de infraestructura de alta complejidad en todo el estado.

**Ethical aspects**

This study used anonymized, aggregated secondary data from publicly available public health information systems, ensuring no individual identification was possible. In accordance with Brazilian National Health Council Resolution No. 510/2016, ethical review and informed consent were waived for this research.

## Introduction

Severe acute respiratory syndrome (SARS) is defined by the onset of two or more symptoms characteristic of influenza-like illness, accompanied by severe symptoms such as respiratory distress (dyspnea), discomfort while breathing, persistent chest pain or pressure, oxygen saturation below 95% in room air, or bluish (cyanosis) of the lips or face, with or without the presence of fever. This definition follows the SARS notification form^1^ ^,^ ^2^. High-risk groups associated with SARS infection and clinical complications include pregnant women, postpartum women, immunocompromised individuals, people under 5 or over 60 years of age, indigenous populations living in villages, and individuals with chronic diseases-primarily linked to living in enclosed or semi-enclosed environments such as households, schools, and daycare centers^3^. In the pediatric population, SARS is one of the leading causes of morbidity and mortality, often requiring admission to pediatric intensive care units, representing a significant public health concern worldwide ^2^.

In Brazil, SARS cases have been mandatorily reported since the 2009 influenza pandemic through the Influenza Epidemiological Surveillance System, including influenza-like illness surveillance in sentinel units. The purpose of reporting is to monitor circulating respiratory viruses and the demand for care due to influenza-like illness ^1^. The impact of COVID-19 on specific populations, including pregnant women and neonates, though still understudied, has been increasingly clarified by recent research. Data related to pregnant women during past outbreaks of coronavirus and Middle East respiratory syndrome (MERS) showed that pregnant women are susceptible to adverse events, such as hospitalization, intensive care unit admission, endotracheal intubation, and acute renal failure ^4^.

Pregnant women are particularly vulnerable to respiratory pathogens and severe pneumonia due to immunological changes and physiological adaptations during pregnancy, including diaphragmatic elevation, increased oxygen consumption, and mucosal edema in the respiratory tract. They are considered a high-risk group for coronavirus-related morbidity and mortality ^5^. Infection caused by the novel coronavirus has become a challenging public health threat worldwide, and data on disease outcomes in pregnant women and neonates remain limited ^6^.

There is scarce information available on coronavirus infection in severely ill pregnant women hospitalized for COVID-19 and their newborns ^7^ ^,^ ^8^. Although case reports of coronavirus infection during pregnancy exist, small sample sizes hinder accurate identification of potential complications ^9^ ^,^ ^10^.

Beyond its direct effects, the COVID-19 pandemic has caused stress and anxiety in pregnant women. Pregnancy-related concerns and stress are associated with clinical conditions such as preeclampsia, depression, increased nausea and vomiting, preterm labor, low Apgar scores, and low birth weight ^5^. This study aimed to investigate whether there was an increase in SARS-related morbidity and mortality among pregnant women and newborns during the first two years of the COVID-19 pandemic in the state of Rio de Janeiro.

## Methods 

Design 

This was a descriptive-analytical study of pregnant women and newborns diagnosed with SARS in the state of Rio de Janeiro, using data from its nine health regions. Cases reported as unspecified SARS, i.e., without a defined etiological agent, were included, as this serves as an indicator of underreporting of COVID-19-related SARS due to the surge in cases during the pandemic ^11^ ^,^ ^12^.

Setting

The state of Rio de Janeiro is divided into nine health regions to improve healthcare organization and service delivery across its municipalities. These regions are part of the Brazilian National Health System (*Sistema Único de Saúde* - SUS) and aim to ensure equitable healthcare access for the state’s population. Each health region has unique characteristics and challenges, reflecting the state’s socioeconomic and geographic diversity. The city of Rio de Janeiro, the state capital, serves as a major healthcare hub, hosting hospitals, research institutions, and specialized medical centers. These facilities serve not only local residents but also act as referral centers for neighboring cities, while smaller municipalities face challenges such as resource limitations and healthcare access disparities ^13^ ^,^ ^14^.

Following the 2009 influenza pandemic in Brazil and the 2020 COVID-19 pandemic, immunization against these viruses has been prioritized. Current recommendations from the World Health Organization, the Centers for Disease Control and Prevention, and professional organizations designate pregnant women, postpartum women, and breastfeeding mothers as priority groups for influenza and COVID-19 vaccination, as they are considered high-risk for SARS, regardless of the etiological agent. In this study, SARS cases and deaths included pregnant women and newborns diagnosed with SARS caused by influenza, COVID-19, or other viruses ^15^.

Participants

Data on SARS notifications in pregnant women and newborns, as well as live birth data from the state of Rio de Janeiro, were used, covering its nine health regions from 2018 to 2021. The nine health regions are: Baía da Ilha Grande, Baixada Litorânea, Centro-Sul, Médio Paraíba, Metropolitana I, Metropolitana II, Noroeste, Norte, and Serrana

Data sources and measurement

Data on pregnant women and newborns (0-27 days) with positive SARS diagnosis from 2018 to 2021 were collected from the Influenza Epidemiological Surveillance Information System (SIVEP-Gripe). Live birth data for 2018-2021 were obtained from the Brazilian Live Birth Information System (SINASC). Case and death data were collected by occurrence in the state of Rio de Janeiro across its nine health regions. For data analysis, two periods were considered: the last two pre-pandemic years (2018-2019) and the first two pandemic years with the highest COVID-19 morbidity and mortality rates in Brazil (2020-2021) ^16^.

Statistical methods

Based on the collected data, frequencies of cases and deaths in newborns and pregnant women were summarized. For pregnant women, case and death frequencies were also analyzed by age group. Morbidity and mortality rates for SARS-along with their averages-were calculated for pregnant women and newborns in the state of Rio de Janeiro and its nine health regions. To obtain the morbidity rate-an indicator used to measure the proportion of pregnant women and newborns diagnosed with SARS in relation to the total population-in pregnant women and newborns, the number of SARS cases in the period was used as the numerator and the number of live births in the period was used as the denominator, multiplied by 100,000 for population standardization ^17^.

The maternal mortality rate measured the risk of maternal death from causes associated with complications during pregnancy, childbirth, and the postpartum period. The newborn mortality rate measured the risk of death between 0 and 27 days after birth. To calculate these two rates, the number of deaths from SARS in the period was used as the numerator and the number of live births in the period was used as the denominator, multiplied by 100,000 for population standardization ^17^.

Subsequently, the Shapiro-Wilk test was used to verify the normality of the variables, and the nonparametric Kruskal-Wallis test-valid for variables that do not follow a normal distribution-was used to compare the difference in frequencies and morbidity and mortality rates between the two periods, before and during the COVID-19 pandemic. Data on SARS cases and deaths in pregnant women and newborns from 2018-2021 were presented. Statistical processing and analysis were performed using IBM SPSS version 20.0 (IBM Corp., Armonk, NY, USA). A 5% significance level was used for comparisons between pre-pandemic and pandemic periods.

## Results

During the COVID-19 pandemic in the state of Rio de Janeiro, there was an increase in SARS cases (n=2,090) and deaths (n=187) among pregnant women compared to pre-pandemic numbers (cases: n=105; deaths: n=8) (Table 1). Similarly, newborns exhibited higher case (n=781) and death (n=36) counts during the pandemic compared to the pre-pandemic period (cases: n=300; deaths: n=8), except in the Baixada Litorânea region, where 31 neonatal cases were recorded during the pandemic versus 56 before. The increase in cases (p-value<0.001) and deaths (p-value<0.001) among pregnant women during the pandemic was statistically significant statewide.

**Table 1:** Frequency of cases and deaths from severe acute respiratory syndrome (SARS) in pregnant women and newborns. State of Rio de Janeiro and its nine health regions, 2018-2019 and 2020-2021

	Pregnant women		Newborns	
	**Cases**	**Deaths**	**Cases**	**Deaths**
	**2018-2019**			
Health region				
Baía da Ilha Grande	4	1	6	0
Baixada Litorânea	3	1	56	0
Centro-Sul	4	0	2	0
Médio Paraíba	8	0	15	0
Metropolitana I	51	4	163	6
Metropolitana II	15	1	17	1
Noroeste	3	0	2	0
Norte	5	1	18	0
Serrana	12	0	21	1
Rio de Janeiro state	105	8	300	8
	**2020-2021**			
Baía da Ilha Grande	19	3	13	1
Baixada Litorânea	96	4	31	1
Centro-Sul	35	6	12	0
Médio Paraíba	92	11	54	2
Metropolitana I	1,289	119	474	21
Metropolitana II	239	22	101	4
Noroeste	18	3	2	0
Norte	123	8	59	3
Serrana	179	11	35	4
Rio de Janeiro state	2,09	187	781	36
p-value^a^	<0.001	<0.001	0.269	0.057

a p-value refers to the state of Rio de Janeiro between the two periods.

Among pregnant women, the 20-29 age group had the highest case frequency before (n=39) and during (n=932) the pandemic, with a statistically significant increase in the pandemic period (p-value<0.001) (Table 2). The 30-39 age group had the second-highest case count (n=802 during the pandemic; n=38 before). The rise in case frequency in these age groups during the pandemic was statistically significant (p-value<0.001).

**Table 2:** Frequencies of cases and deaths from severe acute respiratory syndrome (SARS) in pregnant women by age group. State of Rio de Janeiro and its nine health regions, 2018-2019 and 2020-2021

	10-19 years		20-29 years		30-39 years		40-49 years	
	Cases	Deaths	Cases	Deaths	Cases	Deaths	Cases	Deaths
	2018-2019							
Health region								
Baía da Ilha Grande	0	0	1	0	3	1	0	0
Baixada Litorânea	1	0	1	1	1	0	0	0
Centro-Sul	0	0	2	0	2	0	0	0
Médio Paraíba	4	0	2	0	2	0	0	0
Metropolitana I	10	1	17	2	16	1	8	0
Metropolitana II	3	1	8	0	4	0	0	0
Noroeste	0	0	1	0	2	0	0	0
Norte	1	0	3	1	1	0	0	0
Serrana	1	0	4	0	7	0	0	0
Rio de Janeiro state	20	2	39	4	38	2	8	0
	2020-2021							
Baía da Ilha Grande	0	0	12	2	5	1	2	0
Baixada Litorânea	12	0	50	1	26	2	8	1
Centro-Sul	2	0	12	3	16	3	5	0
Médio Paraíba	5	0	46	4	31	6	10	1
Metropolitana I	127	5	550	34	514	67	98	13
Metropolitana II	28	2	117	8	75	9	19	3
Noroeste	1	0	6	1	8	1	3	1
Norte	11	1	63	5	46	2	3	0
Serrana	13	0	76	4	81	5	9	2
Rio de Janeiro state	199	8	932	62	802	96	157	21
p-value^a^	0.029	0.466	<0.001	<0.001	<0.001	<0.001	<0.001	0.004

a p-value refers to the state of Rio de Janeiro between the two periods.

In 2018-2019, the highest SARS death frequencies among pregnant women were in the 20-29 (n=4) and 30-39 (n=2) age groups (Table 2). During the pandemic (2020-2021), the 30-39 age group had the highest death count (n=96), followed by 20-29 (n=62). This increase in deaths observed during the pandemic period was statistically significant (p-value<0.001). For cases and deaths in pregnant women by age group, metropolitan regions I and II had the highest number of observations before and during the pandemic.

**Figure 1:**
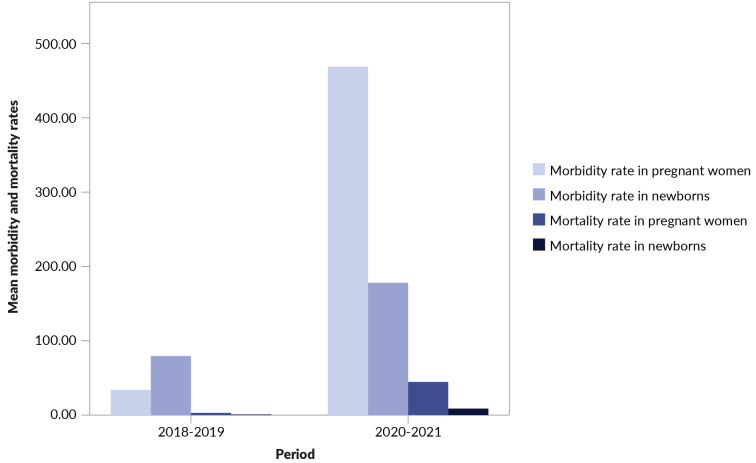
Morbidity and mortality rates from severe acute respiratory syndrome (SARS) in pregnant women and newborns. State of Rio de Janeiro, 2018-2019 and 2020-2021

Morbidity rates in pregnant women increased significantly (p-value<0.001) during the pandemic (537.39/100,000) compared to the pre-pandemic period (24.49/100,000) (Table 3). Mortality rates also showed a statistically significant increase (p-value<0.001) during the pandemic period (48.08/100,000) compared to the pre-pandemic period (1.87/100,000). During the pandemic, mean morbidity rates increased from 32.82/100,000 to 475.53/100,000, and mortality rates rose from 2.88/100,000 to 45.18/100,000 among pregnant women (Figure 1). Before the pandemic, the Ilha Grande Bay region had the highest rates of morbidity (59.92/100,000) and mortality (14.98/100,000) from SARS. During the pandemic, the Serrana region had the highest morbidity rate (819.30/100,000), while Centro-Sul had the highest mortality rate (58.04/100,000).

**Table 3:** Morbidity and mortality rates from severe acute respiratory syndrome (SARS) in pregnant women and newborns. State of Rio de Janeiro and its nine health regions, 2018-2019 and 2020-2021

	Morbidity rate		Mortality rate	
	**Pregnant women**	**Newborns**	**Pregnant women**	**Newborns**
	**2018-2019**			
Health region				
Baía da Ilha Grande	59.92	89.87	14.98	0.00
Baixada Litorânea	14.28	266.55	4.76	0.00
Centro-Sul	39.62	19.81	0.00	0.00
Médio Paraíba	36.55	68.52	0.00	0.00
Metropolitana I	19.55	62.50	1.53	2.30
Metropolitana II	32.15	36.44	2.14	2.14
Noroeste	34.91	23.27	0.00	0.00
Norte	17.43	62.75	3.49	0.00
Serrana	49.30	86.27	0.00	4.11
Rio de Janeiro state	24.49	69.97	1.87	1.87
	**2020-2021**			
Baía da Ilha Grande	312.19	213.60	49.29	16.43
Baixada Litorânea	477.75	154.27	19.91	4.98
Centro-Sul	338.59	116.09	58.04	0.00
Médio Paraíba	455.54	267.38	54.47	9.90
Metropolitana I	550.74	202.52	50.84	8.97
Metropolitana II	567.57	239.85	52.25	9.50
Noroeste	229.45	25.49	38.24	0.00
Norte	466.74	223.88	30.36	11.38
Serrana	819.30	160.20	50.35	18.31
Rio de Janeiro state	537.39	200.82	48.08	9.26
p-value^a^	<0.001	0.022	<0.001	0.008

In newborns, the increase in morbidity rates from 2018-2019 (69.97/100,000) to 2020-2021 (200.82/100,000) was statistically significant (p-value 0.022), as was the rise in mortality rates during the pandemic (9.26/100,000 vs. 1.87/100,000 pre-pandemic; p-value 0.008) (Table 3). Throughout the pandemic, there was an increase in the average morbidity (78.60/100,000 to 180.41/100,000) and mortality (1.04/100,000 to 8.87/100,000) rates in newborns (Figure 1). The Baixada Litorânea region had the highest morbidity rate in the pre-pandemic period (266.55/100,000). During the pandemic, the highest rate was observed in the Médio Paraíba region (267.38/100,000). The Serrana region had the highest mortality rate in newborns in both periods, 2018-2019 (4.11/100,000) and 2020-2021 (18.31/100,000).

## Discussion

This study identified increased frequencies of cases and deaths, as well as higher morbidity-mortality rates from unspecified SARS in pregnant women and newborns in Rio de Janeiro state during the pandemic period compared to the two pre-pandemic years. Such findings demonstrate high incidence and consequent mortality from SARS during the pandemic among pregnant women and newborns, primarily attributable to COVID-19 ^2^ ^,^ ^11^ ^,^ ^18^ ^,^ ^19^.

Both before and during the pandemic, deaths among pregnant women were most frequent in the 30-39 and 20-29 age groups. An elevated risk of death, ICU admission, and invasive ventilation due to SARS was observed with increasing age within the 20-39 age range-whether caused by influenza, COVID-19, or other etiological agents ^18^ ^,^ ^19^.

This study utilized notifications of unspecified SARS (without defined etiological agents) during the pandemic, as these serve as indicators of underreporting of COVID-19-related SARS ^11^ ^,^ ^12^. Importantly, the selection of unspecified SARS was justified not only as an underreporting indicator but also because global influenza cases declined during the pandemic due to reduced testing, social isolation, and potential viral interference ^20^.

It is necessary to discuss regionalization and the particularities of public health in the state of Rio de Janeiro. The public health system in Rio de Janeiro state comprises nine health regions that collectively serve the population. It was observed that Metropolitan Regions I and II showed the greatest increases in the frequency of cases and deaths, as they had the highest population densities in the state and received a migratory flow of individuals seeking care upon experiencing initial symptoms. These two regions also concentrated the largest number of high-complexity healthcare facilities and health professionals ^13^.

The decrease in neonatal morbidity rates in the Baixada Litorânea during the pandemic may have been related to the difficulty in performing diagnostic tests for COVID-19 among the local population, given that this region comprises isolated and poorly connected communities. Similarly, the Northwest Region was characterized by isolated settlements, which hindered access to health services and diagnostic testing, possibly explaining the lack of data before and during the pandemic. This region also had the lowest level of infrastructure and professional training for health surveillance processes ^13^ ^,^ ^14^.

The reduction in morbidity and mortality rates in some health regions of the state from the pre-pandemic to the pandemic period may again be related to population movement toward the Rio de Janeiro metropolitan area during clinical deterioration. In the past decade, state government funding for healthcare was reduced, negatively impacting the quality of care and health surveillance in 2019. It can be inferred that these budget cuts affected the entire state and were exacerbated in 2020 and 2021, the first two years of the COVID-19 pandemic ^21^.

The cases of unspecified SARS during the pandemic, with no defined etiological agent, served as an indicator of underreporting of COVID-19-related SARS cases ^11^ ^,^ ^12^. The findings of this study corroborate those of a multinational cohort involving 2,130 pregnant women across 18 countries, in which those diagnosed with COVID-19 had an increased risk of maternal morbidity and mortality composite index. Neonates born to women with COVID-19 showed high morbidity rates and significantly higher perinatal morbidity and mortality compared to those born to uninfected mothers ^22^.

In the state of Rio de Janeiro, the rise in SARS-related deaths could have been mitigated, particularly in the capital, if there had been better preparation and coverage for managing respiratory syndromes caused by other etiological agents prior to the COVID-19 pandemic-an approach that proved effective in reducing deaths in other locations ^18^. Such an effect was especially evident in the metropolitan region, where access to healthcare and inequities were greater, and the epidemic had a more aggressive course due to the weak local response ^23^. It is essential to note that in developed countries, in addition to socioeconomic and ethno-racial determinants, demographic composition also appeared to influence death rates. Regardless of country or region, there is evidence that the COVID-19 pandemic exposed and deepened existing health inequities, particularly in poorer areas ^24^.

In pregnant women and newborns, SARS due to COVID-19 can be fatal. The timing and mode of delivery should be based on the health condition of the mother and fetus, rather than SARS-CoV-2 infection status or the presence of another etiological agent. Routine separation of mothers with SARS and their newborns is not recommended; however, preventive measures to reduce the risk of transmission should be adopted. The benefits of breastfeeding appear to outweigh the potential risks of viral transmission. Newborns with SARS may present a range of clinical manifestations, from asymptomatic infection to death as the outcome. To reduce the risk of mother-to-child transmission of SARS-causing viruses, the World Health Organization specifically recommends vaccination for pregnant and lactating women ^25^.

This study had certain limitations, including underreporting of cases and deaths on the SIVEP-Gripe system-especially before the COVID-19 pandemic, when, despite mandatory reporting, data entry was inconsistent-and the initial lack of diagnostic tests and exams, which were not widely available. The use of data from mandatory reporting systems such as SIVEP-Gripe provides broad coverage based on census-level data in Brazil. Nevertheless, it may contain inconsistencies due to data entry or form-filling errors, which can affect one or more variables. These notifications undergo continuous review by local health teams, which helps minimize the impact of such errors. 

Due to the increased reporting of unspecified cases during the pandemic, data collection on SARS cases and deaths on SIVEP-Gripe was conducted for all etiological agents, which made it impossible to analyze outcomes by specific causative agent ^11^ ^,^ ^12^ ^,^ ^26^. Data on race/skin color, income, and education level were not used in the study because of poor data quality throughout the state of Rio de Janeiro, both before and during the COVID-19 pandemic.

The COVID-19 pandemic increased SARS-related morbidity and mortality in pregnant women and newborns in the state of Rio de Janeiro during 2020-2021. This study reinforces the understanding that these two groups are particularly vulnerable during infectious disease outbreaks due to altered physiology and compromised immunity. The findings are expected to inform strategies aimed at mitigating harm and to emphasize the importance of conducting epidemiological studies on morbidity and mortality in pregnant women and newborns-especially in Brazil, given its racially diverse population and socioeconomic disparities. It is essential to invest more in harm reduction measures for these groups, such as vaccination, expanded access to prenatal consultations and diagnostic testing for early detection of complications, intensive monitoring of pregnant women at risk for SARS and of morbidity and mortality rates, and improved access to high-complexity equipment and infrastructure throughout the state.

## Data Availability

The data generated during the study are available from the corresponding author upon request
