# Supplementary material for: Increased maternal and neonatal morbidity and mortality due to severe acute respiratory syndrome in the early years of COVID-19: a descriptive-analytical study, Rio de Janeiro state, Brazil, 2018-2021
Source: Epidemiol Serv Saude. 2025 Aug 11;34:e20240665. doi: 10.1590/S2237-96222025v34e20240665.en (PMC12342852; doi:10.1590/S2237-96222025v34e20240665.en)
Supplement: Translation

# Translation: Aumento da morbimortalidade materna e neonatal por síndrome respiratória aguda grave nos primeiros anos de covid-19: estudo descritivo-analítico, estado do Rio de Janeiro, 2018-2021

**DOI:** 10.1590/S2237-96222025v34e20240665.pt

**Authors:** Allan Scharf, Danielle Pereira Paulo, Giselle Ferreira de Paes, Welington Lugão, Danielle Coelho de Azevedo, Beatriz Barde, Nádia Rodrigues

**Affiliations:** 1 Universidade Federal de Alagoas, Instituto de Ciências Biológicas e da Saúde, Maceió, AL, Brasil; 2 Universidade do Estado do Rio de Janeiro, Instituto de Medicina Social, Rio de Janeiro, RJ, Brasil

**Keywords:** Pandemias, Covid-19, Gestantes, Neonatos, Epidemiologia Descritiva

## Abstract

**Objetivo:**

Investigar as taxas de morbidade e mortalidade por síndrome respiratória aguda grave em gestantes e neonatos nos dois primeiros anos da pandemia de covid-19.

**Métodos:**

Tratou-se de estudo descritivo-analítico em que foram coletados dados de casos e óbitos em gestantes e neonatos com diagnóstico positivo para síndrome respiratória aguda grave independentemente do agente etiológico no Sistema de Informação da Vigilância Epidemiológica da Gripe e dados do número de nascidos vivos no Sistema de Informação sobre Nascidos Vivos. Em gestantes e neonatos, calcularam-se as frequências de casos e óbitos e as taxas de morbidade e mortalidade através do teste de Kruskal-Wallis no estado do Rio de Janeiro para comparação entre os períodos 2018-2019 e 2020-2021.

**Resultados:**

Durante a pandemia, observou-se aumento estatisticamente significativo das taxas de morbidade em gestantes (24,49/100 mil para 537,39/100 mil; p-valor<0,001) e neonatos (69,97/100 mil para 200,82/100 mil; p-valor 0,022) e das taxas de mortalidade em gestantes (1,87/100 mil para 48,08/100 mil; p-valor<0,001) e neonatos (1,87/100 mil para 9,26/100 mil; p-valor 0,008).

**Conclusão:**

Este estudo identificou o aumento estatisticamente significativo na pandemia das taxas de morbidade e mortalidade por síndrome respiratória aguda grave em gestantes e neonatos no estado do Rio de Janeiro. Destaca-se a necessidade de maior investimento para a redução de danos nesses grupos, como vacinação e acesso ao sistema de saúde, e de infraestrutura de alta complexidade em todo o estado.

**Aspectos éticos**

Utilizaram-se dados secundários e agregados de sistemas de informação de saúde pública de acesso público, o que impossibilita a identificação individual dos registros. De acordo com a Resolução do Conselho Nacional de Saúde n° 510/2016, este estudo dispensa apreciação por comitê de ética em pesquisa e uso do registro de consentimento livre e esclarecido.

## Introdução

A síndrome respiratória aguda grave (SRAG) é definida pelo surgimento de dois ou mais sintomas respiratórios característicos da síndrome gripal, acompanhados de sintomas de gravidade, como dificuldade respiratória (dispneia), desconforto ao respirar, dor ou pressão persistente no tórax, saturação de oxigênio inferior a 95% em ar ambiente ou coloração azulada (cianose) nos lábios ou no rosto, com ou sem presença de febre. Essa definição segue a ficha de notificação de SRAG ^1^ ^,^ ^2^. Os grupos de risco associados à infecção por SRAG e complicações clínicas são gestantes, puérperas, imunossuprimidos, pessoas com idade menor que 5 anos ou maior que 60 anos, indígenas aldeados e pessoas com doença crônica, relacionados principalmente à vivência em ambientes fechados ou semifechados, como o domicílio, escolas e creches ^3^. Na população pediátrica, é uma das principais causas de morbidade e mortalidade, muitas vezes sendo necessária internação na unidade de terapia intensiva pediátrica, o que representa um problema para a saúde pública mundial ^2^.

No Brasil, é obrigatória a notificação dos casos de SRAG desde a pandemia de influenza de 2009, através do Sistema de Vigilância Epidemiológica da influenza, incluindo a vigilância de síndrome gripal em unidades sentinelas. A finalidade da notificação é de monitorar os vírus respiratórios circulantes e a demanda de atendimentos por síndrome gripal ^1^. O impacto da covid-19 em populações específicas, incluindo gestantes e neonatos, apesar de ainda pouco estudado, tem sido esclarecido por crescentes trabalhos. Dados relacionados a mulheres grávidas durante surtos passados de coronavírus e síndrome respiratória do oriente médio mostraram que gestantes são suscetíveis a experimentar eventos adversos, como necessidade de hospitalização ou internação em unidade de terapia intensiva, intubação endotraqueal e insuficiência renal aguda ^4^.

As gestantes são particularmente suscetíveis aos patógenos respiratórios e às pneumonias graves devido às alterações imunológicas e adaptações fisiológicas durante a gestação, como a elevação do diafragma, o aumento do consumo de oxigênio e o aparecimento de edema da mucosa do trato respiratório. As mulheres grávidas são consideradas um dos principais grupos de risco para a morbidade e mortalidade pelos coronavírus ^5^. A infecção causada pelo novo coronavírus tornou-se ameaça desafiadora à saúde pública em todo o mundo, e, quando se trata de gestantes e neonatos, os dados sobre os desfechos da doença são limitados ^6^.

Existem poucas informações disponíveis sobre a infecção por coronavírus em gestantes gravemente doentes hospitalizadas por covid-19 e seus filhos recém-nascidos ^7^ ^,^ ^8^. Há relatos de casos de infecção por coronavírus durante a gravidez, mas o pequeno tamanho da amostra dificulta a localização adequada de possíveis complicações ^9^ ^,^ ^10^.

A pandemia de covid-19, além dos efeitos diretos, produziu estresse e ansiedade nas mulheres grávidas. A preocupação e o estresse na gravidez estão presentes em condições clínicas como pré-eclâmpsia, depressão, aumento de náuseas e vômitos, trabalho de parto prematuro, baixo Apgar e peso ao nascer do bebê ^5^. Este estudo teve como objetivo investigar se houve aumento da morbimortalidade por SRAG em gestantes e neonatos nos dois primeiros anos da pandemia de covid-19 no estado do Rio de Janeiro.

## Métodos 

Delineamento 

Tratou-se de estudo descritivo-analítico em gestantes e neonatos diagnosticados por SRAG no estado do Rio de Janeiro, por meio de informações sobre suas nove regiões de saúde. Utilizou-se a notificação por SRAG não especificada, ou seja, sem agente etiológico definido, pois esta é um indicador da subnotificação de SRAG por covid-19 causada pelo aumento de casos durante a pandemia ^11^ ^,^ ^12^.

Contexto

O estado do Rio de Janeiro é dividido em nove regiões de saúde, o que objetiva aprimorar a organização e a prestação de serviços de saúde em seus municípios. Essas regiões fazem parte do Sistema Único de Saúde e visam garantir acesso equitativo à saúde para a população do estado. Cada região de saúde tem suas próprias características e desafios únicos, o que reflete a diversidade socioeconômica e geográfica do estado. A cidade do Rio de Janeiro, a capital do estado, serve como importante centro de serviços de saúde, pois abriga hospitais, instituições de pesquisa e centros médicos especializados. Essas instalações não atendem apenas aos moradores locais, mas também servem como centros de referência para cidades vizinhas, enquanto municípios menores enfrentam desafios como limitações de recursos e disparidades no acesso à saúde ^13^ ^,^ ^14^.

Após a pandemia de influenza no Brasil em 2009 e a pandemia de covid-19 em 2020, a imunização contra esses vírus tem sido prioridade. As recomendações atuais da Organização Mundial da Saúde, dos Centros de Controle e Prevenção de Doenças e de organizações profissionais são para que gestantes, puérperas e lactantes recebam prioridade para a vacinação contra influenza e covid-19, pois essas mulheres foram designadas como grupo de risco para SRAG, independentemente do agente etiológico. Neste estudo, foram considerados como casos e óbitos por SRAG gestantes e neonatos diagnosticados por SRAG - causada por influenza, covid-19 ou outro vírus ^15^.

Participantes

Utilizaram-se dados de notificação por SRAG em gestantes e neonatos e dados de nascidos vivos do estado do Rio de Janeiro, por meio de informações sobre suas nove regiões de saúde no período 2018-2021. As nove regiões de saúde que compõem o estado do Rio de Janeiro são: Baía da Ilha Grande, Baixada Litorânea, Centro-Sul, Médio Paraíba, Metropolitana I, Metropolitana II, Noroeste, Norte e Serrana.

Fontes de dados e mensuração

Os dados de gestantes e neonatos (0-27 dias) que tiveram diagnóstico positivo para SRAG de 2018 a 2021 foram coletados dos bancos de dados do Sistema de Informação da Vigilância Epidemiológica da Gripe (SIVEP-Gripe). Os dados do número de nascidos vivos para o período 2018-2021 foram coletados no Sistema de Informação sobre Nascidos Vivos. Os dados dos casos e óbitos foram coletados por ocorrência no estado do Rio de Janeiro em suas nove regiões de saúde. Para a análise dos dados, foram considerados dois períodos: os dois últimos anos pré-pandêmicos (2018 e 2019) e os dois primeiros anos pandêmicos e com maiores taxas de morbidade e mortalidade por covid-19 no Brasil (2020 e 2021) ^16^.

Métodos estatísticos

Com base nos dados obtidos, foram sumarizadas as frequências de casos e óbitos nos neonatos e em gestantes. Em gestantes, as frequências de casos e óbitos foram analisadas também por faixa etária. Calcularam-se as taxas de morbidade e mortalidade por SRAG e suas médias em gestantes e neonatos do estado do Rio de Janeiro e de suas nove regiões de saúde. Para obtenção da taxa de morbidade - indicador para medir a proporção de gestantes e neonatos diagnosticados com SRAG em relação à população total - em gestantes e neonatos, foi utilizado como numerador o número de casos de SRAG no período e como denominador o número de nascidos vivos no período, multiplicado por 100 mil para padronização populacional ^17^.

A taxa de mortalidade em gestantes mediu o risco de morte materna em decorrência de causas associadas a complicações durante a gestação, o parto e o puerpério. A taxa de mortalidade em neonatos mediu o risco de morte de 0 a 27 dias após o nascimento. Para calcular essas duas taxas, foi utilizado o número de óbitos por SRAG no período no numerador e, no denominador, o número de nascidos vivos no período, multiplicado por 100 mil para padronização entre as populações ^17^.

Em seguida, utilizaram-se o teste Shapiro-Wilk, para verificar a normalidade das variáveis, e o teste não paramétrico Kruskal-Wallis, válido para variáveis que não seguem distribuição normal, para comparar a diferença das frequências e das taxas de morbidade e mortalidade entre os dois períodos, antes e durante a pandemia de covid-19. Os valores referentes ao número de casos e de óbitos por SRAG em gestantes e neonatos no período 2018-2021 foram apresentados. Para realizar o processamento e a análise estatística, utilizou-se o software IBM SPSS versão 20.0 (IBM Corp., Armonk, NY, USA). Considerou-se o nível de significância estatística de 5% para as análises de comparação da diferença de frequências e taxas entre os períodos pré-pandêmico e pandêmico.

## Resultados

Durante a pandemia no estado do Rio de Janeiro, houve aumento no número de casos (n=2.090) e óbitos (n=187) observados por SRAG em gestantes comparado com o número de casos (n=105) e óbitos (n=8) antes da pandemia (Tabela 1). O número de casos (n=781) e óbitos (n=36) observados também foi maior em neonatos no período pandêmico comparado ao número de casos (n=300) e óbitos (n=8) no período pré-pandêmico, com exceção de casos em neonatos na região da Baixada Litorânea, em que foram observados 31 casos durante a pandemia e 56 casos antes da pandemia. Durante a pandemia, o aumento nos números de casos (p-valor<0,001) e óbitos (p-valor<0,001) em gestantes foi estatisticamente significativo no estado.

**Tabela 1:** Frequências de casos e óbitos por síndrome respiratória aguda grave (SRAG) em gestantes e neonatos. Estado do Rio de Janeiro e suas nove regiões de saúde, 2018-2019 e 2020-2021

	Gestantes		Neonatos	
	Casos	Óbitos	Casos	Óbitos
	Período 2018-2019			
Região de saúde				
Baía da Ilha Grande	4	1	6	0
Baixada Litorânea	3	1	56	0
Centro-Sul	4	0	2	0
Médio Paraíba	8	0	15	0
Metropolitana I	51	4	163	6
Metropolitana II	15	1	17	1
Noroeste	3	0	2	0
Norte	5	1	18	0
Serrana	12	0	21	1
Estado do Rio de Janeiro	105	8	300	8
	Período 2020-2021			
Baía da Ilha Grande	19	3	13	1
Baixada Litorânea	96	4	31	1
Centro-Sul	35	6	12	0
Médio Paraíba	92	11	54	2
Metropolitana I	1.289	119	474	21
Metropolitana II	239	22	101	4
Noroeste	18	3	2	0
Norte	123	8	59	3
Serrana	179	11	35	4
Estado do Rio de Janeiro	2.090	187	781	36
p-valor^a^	<0,001	<0,001	0,269	0,057

a p-valor referente ao estado do Rio de Janeiro entre os dois períodos.

Em gestantes, a faixa etária 20-29 anos apresentou a maior frequência de casos antes da pandemia (n=39) e durante a pandemia (n=932), com aumento estatisticamente significativo no período pandêmico (p-valor<0,001) (Tabela 2). A faixa etária de 30-39 anos foi a segunda com mais casos (n=802 durante a pandemia; n=38 antes da pandemia). O aumento da frequência de casos nessas faixas etárias no período de pandemia em relação ao período antes da pandemia foi estatisticamente significativo (p-valor<0,001).

**Tabela 2:** Frequências de casos e óbitos por síndrome respiratória aguda grave (SRAG) em gestantes por faixa etária. Estado do Rio de Janeiro e suas nove regiões de saúde, 2018-2019 e 2020-2021

	10-19 anos		20-29 anos		30-39 anos		40-49 anos	
	Casos	Óbitos	Casos	Óbitos	Casos	Óbitos	Casos	Óbitos
	Período 2018-2019							
Região de saúde								
Baía da Ilha Grande	0	0	1	0	3	1	0	0
Baixada Litorânea	1	0	1	1	1	0	0	0
Centro-Sul	0	0	2	0	2	0	0	0
Médio Paraíba	4	0	2	0	2	0	0	0
Metropolitana I	10	1	17	2	16	1	8	0
Metropolitana II	3	1	8	0	4	0	0	0
Noroeste	0	0	1	0	2	0	0	0
Norte	1	0	3	1	1	0	0	0
Serrana	1	0	4	0	7	0	0	0
Estado do Rio de Janeiro	20	2	39	4	38	2	8	0
	Período 2020-2021							
Baía da Ilha Grande	0	0	12	2	5	1	2	0
Baixada Litorânea	12	0	50	1	26	2	8	1
Centro-Sul	2	0	12	3	16	3	5	0
Médio Paraíba	5	0	46	4	31	6	10	1
Metropolitana I	127	5	550	34	514	67	98	13
Metropolitana II	28	2	117	8	75	9	19	3
Noroeste	1	0	6	1	8	1	3	1
Norte	11	1	63	5	46	2	3	0
Serrana	13	0	76	4	81	5	9	2
Estado do Rio de Janeiro	199	8	932	62	802	96	157	21
p-valor^a^	0,029	0,466	<0,001	<0,001	<0,001	<0,001	<0,001	0,004

a p-valor referente ao estado do Rio de Janeiro entre os dois períodos.

As faixas etárias em gestantes que apresentaram maior frequência de óbitos por SRAG no período 2018-2019 foram 20-29 anos (n=4) e 30-39 anos (n=2) (Tabela 2). Durante a pandemia, no período 2020-2021, a faixa etária com maior frequência de óbitos foi 30-39 anos (n=96), seguida por 20-29 anos (n=62). Esse aumento de óbitos observado no período pandêmico foi estatisticamente significativo (p-valor<0,001). Para casos e óbitos em gestantes por faixa etária, as regiões metropolitanas I e II apresentaram o maior número de observações antes e durante a pandemia.

As taxas de morbidade em gestantes aumentaram significativamente (p-valor<0,001) no período pandêmico (537,39/100 mil) em relação ao período pré-pandêmico (24,49/100 mil) (Tabela 3). As taxas de mortalidade também apresentaram aumento estatisticamente significativo (p-valor<0,001) durante o período de pandemia (48,08/100 mil) comparadas ao período pré-pandemia (1,87/100 mil). Durante a pandemia, observou-se aumento nas médias das taxas de morbidade (32,82/100 mil para 475,53/100 mil) e mortalidade (2,88/100 mil para 45,18/100 mil) em gestantes (Figura 1). A região da Baía da Ilha Grande antes da pandemia apresentou as maiores taxas de morbidade (59,92/100 mil) e mortalidade (14,98/100 mil) por SRAG. Durante a pandemia, observaram-se a maior taxa de morbidade (819,30/100 mil) na região Serrana e a maior taxa de mortalidade na região Centro-Sul (58,04/100 mil).

**Tabela 3:** Taxas de morbidade e mortalidade por síndrome respiratória aguda grave (SRAG) em gestantes e neonatos. Estado do Rio de Janeiro e suas nove regiões de saúde, 2018-2019 e 2020-2021

	Taxa de morbidade		Taxa de mortalidade	
	Gestantes	Neonatos	Gestantes	Neonatos
	2018-2019			
Região de saúde				
Baía da Ilha Grande	59,92	89,87	14,98	0
Baixada Litorânea	14,28	266,55	4,76	0
Centro-Sul	39,62	19,81	0	0
Médio Paraíba	36,55	68,52	0	0
Metropolitana I	19,55	62,5	1,53	2,3
Metropolitana II	32,15	36,44	2,14	2,14
Noroeste	34,91	23,27	0	0
Norte	17,43	62,75	3,49	0
Serrana	49,3	86,27	0	4,11
Estado do Rio de Janeiro	24,49	69,97	1,87	1,87
	2020-2021			
Baía da Ilha Grande	312,19	213,6	49,29	16,43
Baixada Litorânea	477,75	154,27	19,91	4,98
Centro-Sul	338,59	116,09	58,04	0
Médio Paraíba	455,54	267,38	54,47	9,9
Metropolitana I	550,74	202,52	50,84	8,97
Metropolitana II	567,57	239,85	52,25	9,5
Noroeste	229,45	25,49	38,24	0
Norte	466,74	223,88	30,36	11,38
Serrana	819,3	160,2	50,35	18,31
Estado do Rio de Janeiro	537,39	200,82	48,08	9,26
p-valor^a^	<0,001	0,022	<0,001	0,008

a p-valor referente ao estado do Rio de Janeiro entre os dois períodos.

Em neonatos, o aumento da taxa de morbidade no período 2020-2021 (200,82/100 mil) em relação ao período 2018-2019 (69,97/100 mil) foi estatisticamente significativo (p-valor 0,022), assim como o aumento da taxa de mortalidade durante a pandemia (9,26/100 mil) comparado à antes da pandemia (1,87/100 mil) (p-valor 0,008) (Tabela 3). Ao longo da pandemia, observou-se aumento nas médias das taxas de morbidade (78,60/100 mil para 180,41/100 mil) e mortalidade (1,04/100 mil para 8,87/100 mil) em neonatos (Figura 1). A região da Baixada Litorânea apresentou a maior taxa de morbidade no período pré-pandêmico (266,55/100 mil). No período pandêmico, a maior taxa foi observada na região do Médio Paraíba (267,38/100 mil). A região Serrana apresentou a maior taxa de mortalidade em neonatos em ambos os períodos, 2018-2019 (4,11/100 mil) e 2020-2021 (18,31/100 mil).

## Discussão

Este estudo identificou o aumento das frequências de casos e óbitos e das taxas de morbimortalidade por SRAG não especificada em gestantes e neonatos no estado do Rio de Janeiro durante o período pandêmico em comparação com os últimos dois anos pré-pandêmicos. Estes achados mostraram alta incidência e, consequentemente, óbito por SRAG durante a pandemia em gestantes e neonatos, causados principalmente pela covid-19 ^2^ ^,^ ^11^ ^,^ ^18^ ^,^ ^19^.

**Figura 1:** Taxas de morbidade e mortalidade por síndrome respiratória aguda grave (SRAG) em gestantes e neonatos. Estado do Rio de Janeiro, 2018-2019 e 2020-2021

Antes e durante a pandemia, o número de óbitos em gestantes apresentou maior frequência em gestantes nas faixas etárias de 30-39 anos e 20-29 anos. Observou-se risco aumentado de óbito, admissão em unidade de terapia intensiva e ventilação invasiva por SRAG quanto maior fosse a idade na faixa etária de 20-39 anos - causada por influenza, covid-19 ou outros agentes etiológicos ^18^ ^,^ ^19^.

Este estudo utilizou as notificações por SRAG não especificada durante a pandemia, sem agente etiológico definido, pois estas são indicador da subnotificação de SRAG causada pela covid-19 ^11^ ^,^ ^12^. É importante ressaltar que a escolha pela SRAG não especificada, além de ser indicador de subnotificação, se deu porque, durante a pandemia, foi observada a diminuição nos casos de influenza globalmente, ocorrida pela menor testagem, pelo isolamento social ou até mesmo pela competição fisiológica entre os vírus ^20^.

É preciso discutir a regionalização e as particularidades da saúde pública no estado do Rio de Janeiro. O estado conta com nove regiões de saúde que, somadas, contribuem para o acesso à saúde da população que reside no território estadual. Observou-se que as regiões metropolitanas I e II apresentaram o maior aumento nas frequências de casos e óbitos, pois elas tinham as maiores densidades demográficas do estado, além de haver fluxo migratório até essas regiões ao sinal dos primeiros sintomas. Essas duas regiões concentraram o maior quantitativo de equipamentos de alta complexidade e profissionais de saúde ^13^.

A diminuição na taxa de morbidade em neonatos na Baixada Litorânea durante a pandemia pôde estar relacionada à dificuldade em realizar testes diagnósticos de covid-19 em seus habitantes, por essa região apresentar povoações isoladas e pouco articuladas entre si. Assim como a Baixada Litorânea, a região Noroeste possuía povoações isoladas, dificultando a busca por atendimentos e a realização de diagnósticos, o que pode explicar a falta de dados antes e durante a pandemia. A região Noroeste possuía menor infraestrutura e qualificação profissional para os processos de vigilância em saúde ^13^ ^,^ ^14^.

A diminuição das taxas de morbidade e mortalidade em regiões de saúde do estado do momento pré-pandêmico para o pandêmico pôde estar relacionada novamente com o deslocamento da população para a região metropolitana do estado do Rio de Janeiro durante a pandemia, especialmente em situações em que houve o agravamento da condição clínica. Na última década, houve redução do financiamento da atenção à saúde pelo governo estadual, o que impactou a qualidade da assistência e da vigilância em saúde em 2019. Pode-se inferir que essas reduções orçamentárias tiveram impacto em todo o estado, agravado em 2020 e 2021, os primeiros dois anos da pandemia de covid-19 ^21^.

Os casos de SRAG não especificados durante a pandemia, sem agente etiológico definido, foram indicador da subnotificação de SRAG causada pela covid-19 ^11^ ^,^ ^12^. Os resultados apresentados neste estudo corroboram a coorte multinacional, na qual foram investigadas 2.130 gestantes em 18 países, em que as mulheres com diagnóstico de covid-19 apresentavam risco aumentado de índice composto de morbidade e mortalidade materna. Neonatos de mulheres com diagnóstico de covid-19 apresentaram alto índice de morbidade, além de índice de morbimortalidade perinatal significativamente maiores em comparação com neonatos de mulheres sem diagnóstico de covid-19 ^22^.

No estado do Rio de Janeiro, o cenário de aumento de óbitos por SRAG poderia ter sido atenuado se houvesse, principalmente na capital, a melhor preparação e cobertura para lidar com síndromes respiratórias causadas por outros agentes etiológicos antes do começo da pandemia por covid-19, o que se mostrou eficaz e diminuiu o número de óbitos em outros locais durante a pandemia ^18^. Pôde-se observar esse efeito especialmente na região metropolitana, onde o acesso à saúde e as iniquidades são aumentados e a epidemia se comportou de forma mais agressiva pela fraca resposta local ^23^. É fundamental ressaltar que, em países desenvolvidos, além dos determinantes socioeconômicos e étnico-raciais, a composição demográfica das populações também pareceu influenciar no número de óbitos. Pode-se inferir que, independentemente do país e da região, há evidências de que a pandemia de covid-19 expôs e ampliou as iniquidades em saúde, principalmente nas regiões mais pobres ^24^.

Em gestantes e neonatos, casos de SRAG por covid-19 podem ser fatais. O momento e o modo de parto devem ser decididos pelo estado de saúde da mãe e do feto em vez da positividade para coronavírus ou outro agente etiológico. A separação rotineira de mães com SRAG e seus bebês não é recomendada, no entanto é importante tomar medidas preventivas para reduzir o risco de transmissão. As vantagens da amamentação parecem superar os perigos potenciais da transmissão viral. Neonatos com quadros de SRAG podem apresentar diferentes manifestações clínicas, desde infecções assintomáticas até desfecho de óbito. Para que se diminua o risco de transmissão de vírus causadores de SRAG da mãe para o filho, a Organização Mundial da Saúde recomenda especificamente a vacinação em gestantes e lactantes ^25^.

Este estudo teve como limitações a subnotificação de casos e óbitos no sistema SIVEP-Gripe - principalmente antes da pandemia por covid-19, quando, apesar de ser obrigatória a notificação, não era feita de forma consistente - e a falta de testes e exames diagnósticos no começo da pandemia, que não estavam amplamente disponíveis. O uso de dados originados de sistemas de notificação obrigatória, como o SIVEP-Gripe, oferece ampla cobertura por se basear em informações censitárias do Brasil. No entanto, pode apresentar eventuais inconsistências devido a erros de digitação ou preenchimento, o que afeta um ou mais campos do registro. As notificações passam por revisões contínuas realizadas pelas equipes locais responsáveis, o que reduz o impacto dessas possíveis falhas. 

Por causa do aumento da notificação de casos não especificados devido à pandemia, a coleta de dados de casos e óbitos por SRAG no SIVEP-Gripe por todos os agentes etiológicos foi preferida, o que inviabilizou a avaliação de casos e óbitos separada por agente causador ^11^ ^,^ ^12^ ^,^ ^26^. Não foram utilizados dados de cor/raça da pele, renda e escolaridade no estudo, em razão da baixa qualidade com que esses dados são preenchidos em todo o estado do Rio de Janeiro, antes e durante a pandemia por covid-19.

A pandemia de covid-19 aumentou a morbimortalidade por SRAG em gestantes e neonatos no estado do Rio de Janeiro durante o período 2020-2021. Este estudo corrobora que esses dois grupos são considerados vulneráveis em surtos de doenças infecciosas devido à fisiologia alterada e à imunidade comprometida. Espera-se que os dados apresentados contribuam para estratégias com o objetivo de reduzir danos, reiterando a importância de estudos epidemiológicos de morbimortalidade em gestantes e neonatos especialmente no Brasil, por sua população miscigenada e característica socioeconômica desigual. Destaca-se a necessidade de maior investimento para a redução de danos nesses grupos, como vacinação, expansão do acesso a consultas e exames pré-natais para detecção precoce de complicações, monitoramento intensivo de gestantes com fatores de risco para SRAG e das taxas de morbidade e mortalidade, e infraestrutura de equipamentos de alta complexidade em todo o estado

## Data Availability

Os dados gerados durante o estudo estão disponíveis com o autor correspondente, mediante pedido
